# A luteinizing hormone receptor intronic variant is significantly associated with decreased risk of Alzheimer's disease in males carrying an apolipoprotein E ε4 allele

**DOI:** 10.1186/1471-2350-9-37

**Published:** 2008-04-25

**Authors:** Ryan J Haasl, M Reza Ahmadi, Sivan Vadakkadath Meethal, Carey E Gleason, Sterling C Johnson, Sanjay Asthana, Richard L Bowen, Craig S Atwood

**Affiliations:** 1 Section of Geriatrics and Gerontology, Department of Medicine, University of Wisconsin School of Medicine and Public Health, Madison, WI 53705, USA; 2 Geriatric Research, Education and Clinical Center (GRECC), Williams S. Middleton Memorial Veterans Hospital, Madison, WI 53705, USA; 3 OTB Research, Charleston, SC, 29464, USA; 4 School of Exercise, Biomedical and Health Sciences, Edith Cowan University, Joondalup, WA 6027, Australia

## Abstract

Genetic and biochemical studies support the apolipoprotein E (*APOE*) ε4 allele as a major risk factor for late-onset Alzheimer's disease (AD), though ~50% of AD patients do not carry the allele. APOE transports cholesterol for luteinizing hormone (LH)-regulated steroidogenesis, and both LH and neurosteroids have been implicated in the etiology of AD. Since polymorphisms of LH beta-subunit (*LHB*) and its receptor (*LHCGR*) have not been tested for their association with AD, we scored AD and age-matched control samples for *APOE *genotype and 14 polymorphisms of *LHB *and *LHCGR*. Thirteen gene-gene interactions between the loci of *LHB*, *LHCGR*, and *APOE *were associated with AD. The most strongly supported of these interactions was between an *LHCGR *intronic polymorphism (rs4073366; lhcgr2) and *APOE *in males, which was detected using all three interaction analyses: linkage disequilibrium, multi-dimensionality reduction, and logistic regression. While the *APOE *ε4 allele carried significant risk of AD in males [p = 0.007, odds ratio (OR) = 3.08(95%confidence interval: 1.37, 6.91)], ε4-positive males carrying 1 or 2 C-alleles at lhcgr2 exhibited significantly decreased risk of AD [OR = 0.06(0.01, 0.38); p = 0.003]. This suggests that the lhcgr2 C-allele or a closely linked locus greatly reduces the risk of AD in males carrying an *APOE *ε4 allele. The reversal of risk embodied in this interaction powerfully supports the importance of considering the role gene-gene interactions play in the etiology of complex biological diseases and demonstrates the importance of using multiple analytic methods to detect well-supported gene-gene interactions.

## Background

Alzheimer's disease (AD) is a progressive neurodegenerative disorder characterized by neuronal and synaptic loss, neurofibrillary tangles in neuronal cytoplasm, and deposition of β-amyloid (Aβ) in extracellular, neuritic plaques. To date, only four genes have been unambiguously associated with AD, of which only one, Apolipoprotein E (*APOE*), is associated with the common, late-onset form of AD [1]. The *APOE4 *allele (ε4) was first identified as a risk factor for late-onset AD in the early 1990s [2,3], and corroborated as such by a number of subsequent studies [4]. However, the risk for AD imparted by one or two ε4 alleles is only partially penetrant: ~50% of AD patients do not carry an ε4 allele [5]. Application of quantitative genetics methodology in fact supports the presence of 4 as yet unidentified AD-associated loci in the human genome, each expected to affect age of onset (AoO) as much or more than *APOE *[6]. Additional genetic risk factors for AD, therefore, remain to be found. Yet, a majority of studies have failed to find any evidence for association of their genetic target(s) with AD (e.g., recently, Chapuis et al. [7] and [8]), and large-scale meta-analyses, which combine the datasets of numerous studies, often negate or call into question any putative associations inferred from individual datasets [9].

The disproportionate number of women who suffer from AD has long suggested that an aspect of reproductive physiology lies at the origin of AD pathogenesis. Recently, this idea was supported by the discovery that polymorphisms of the estrogen receptors alpha and beta were associated with AD, further implicating estradiol signaling in the pathogenesis of AD [10,11]. Several converging lines of evidence make another member of the hypothalamic-pituitary-gonadal axis, luteinizing hormone (LH), a worthwhile candidate for genetic study: (1) LH is elevated in AD patients [12-14]; (2) LH crosses the blood-brain barrier [15]; (3) in the brain, LH/chorionic gonadotropin receptors (LHCGR) are most concentrated in the hippocampus [16]; (4) increased concentration of LH has been shown to increase Aβ secretion in a neuronal cell line while suppression of serum LH decreases brain Aβ in mice [17]; and, (5) reduced serum LH has been shown to decrease cognitive loss and Aβ deposition in AβPP transgenic mice [18]. Interestingly, through its regulation of steroidogenic enzymes, LH mediates neurosteroid production from cholesterol [19]; both animal and human clinical studies strongly support the crucial neuroprotective functions of steroids in the brain [20,21]. Since APOE is a cholesterol transport protein [22] involved in the transport of cholesterol into neurons [23] for neurosteroid synthesis, a functional link exists between *APOE *and LH signaling.

Numerous polymorphisms of LH beta-subunit (*LHB*) and *LHCGR *have been documented (for comprehensive reviews, see [24] and [25]). While the majority of mutations underlying these polymorphisms are associated with rare reproductive disorders, a few are relatively more common and worthy of exploring for their association with AD. Two non-synonymous single nucleotide polymorphisms (SNPs) in *LHB *are collectively referred to as variant LH (vLH) [26]. In a study of 40 Japanese women, vLH carriers exhibited greater LH secretion in response to GnRH stimulation [27]. In breast cancer patients, an LQ-insert in exon 1 of *LHCGR *was associated with a significantly earlier age of onset and worse survival rate [27]. Exon 10 of *LHCGR *is required for binding of LH [28] and is the location of 2 relatively common non-synonymous SNPs [29]. The functional consequences of the mutations underlying the other *LHB *and *LHCGR *polymorphisms scored in our study, however, are largely unknown. Therefore, in this study we examined polymorphmic sites of LH β-subunit (*LHB*) and *LHCGR*, as well as gene-gene interactions between *LHB*, *LHCGR*, and *APOE *for association with AD. Our results suggest that a specific LHCGR allele significantly decreases the risk of AD in individuals carrying an APOE ε4 allele.

## Results

*APOE *genotype and 14 previously reported polymorphisms of *LHB *and *LHCGR *were scored (Table 1). The A/G polymorphism in exon 2 of *LHB *(rs5030775) was invariant and, therefore, not included in any analyses. The 2 SNPs comprising vLH (rs1800447) covaried without fail, as did the *LHB *polymorphisms rs2387588 and rs4287687; in both instances, only one of the polymorphisms was subjected to analysis. Table 1 lists the names assigned to the remaining 11 polymorphisms of *LHB *and *LHCGR*.

**Table 1:** Scored polymorphisms of *LHB *and *LHCGR*.

**Gene**	**Designation**	**dbSNP reference ID**	**Location**	**Type**
***LHB***	lhb1	rs3956233	Intron 1	Intronic SNP
	lhb2	rs4002462	Intron 1	Intronic SNP
	*	rs5030775^a^	Exon 2 (signal peptide)	Non-synonymous SNP
	lhb3	rs1800447	Exon 2 (vLH SNP 1)	Non-synonymous SNP
	**	(rs1800447)^b^	Exon 2 (vLH SNP 2)	Non-synonymous SNP
	lhb4	rs6521	Exon 2	Synonymous SNP
	lhb5	rs1056914	Exon 2	Synonymous SNP
	lhb6	rs2387588	Intron 2	Intronic SNP
	lhb7	rs4287687	Intron 2	Intronic SNP
***LHR***	lhcgr1	rs4539842	Exon 1	6 base insertion/deletion
	lhcgr2	rs4073366	Intron 1	Intronic SNP
	lhcgr3	rs12470652	Exon 10	Non-synonymous SNP
	lhcgr4	rs2293275	Exon 10	Non-synonymous SNP
	lhcgr5	rs13006488	Exon 11	Synonymous SNP

^a^rs5030775 not included in analyses, as it was invariant in our cohort

^b^second vLH position not included in analyses since it is in complete linkage disequilibrium with lhb3

### Analysis of single-locus, main effects

#### HWE and AoO

**Figure 1:**
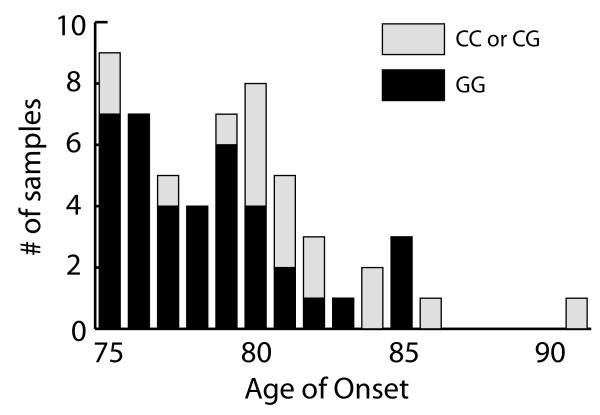
**Age of onset is significantly affected by lhcgr2 genotype in males**. AoO in lhcgr2 heterozygous and homozygous males (n = 50, p = 0.001).

The only locus demonstrating significant divergence from HWE at the modified FDR level was lhcgr3 in the control (C) group (AD: p = 0.052, C: p = 0.008; α = 0.0082). This result is apparently driven by samples of the female control group (Cf; p = 0.034), as the male control group (Cm) is not even marginally divergent from HWE (p = 0.149). No main or interactive effects identified in our analyses included lhcgr3. Only one genotype model was significantly associated with age of onset (AoO) at the modified FDR level. In the ADm group, AoO was affected by lhcgr2 genotype (Figure 1). Males with 1 or 2 C-alleles of lhcgr2 had a mean AoO of 81.06 years, while males with no C-allele had a mean AoO of 78.33 (n = 50, p = 0.0047; α = 0.0077) – i.e., the C-allele significantly delays AoO in males.

#### χ ^2^-tests for single-locus associations

Whether stratified by gender or not, no significant associations between *LHB *or *LHCGR *loci and AD were identified. As expected, the frequency of the *APOE *ε4 allele was much greater in AD than in C samples: 0.35 and 0.09, respectively. ε4 was also found at a higher frequency in AD females (ADf; 0.39) than in AD males (ADm; 0.32). Compared with ADm, a noticeably greater number of ε4 alleles were found in ADf *heterozygotes *(ε2/ε4 and ε3/ε4: 0.35 in ADm, 0.62 in ADf) than in *homozygotes *(ε4/ε4: 0.14 in ADm, 0.08 in ADf). A significant association, at modified FDR levels, between the *APOE *ε4 allele and AD was detected in AD vs. C (p < 0.0001; α = 0.0082), ADm vs. Cm (p < 0.001; α = 0.0077), and ADf vs. C female (Cf; p < 0.0001; α = 0.0081) comparisons. Both the 'ε4 dosage' and 'ε4 positive' models of *APOE *genotype were associated with AD at marginally to highly significant levels in AD vs. C (p < 0.0001; α = 0.0082), ADm vs. Cm (ε4 dosage: p = 0.003; ε4 positive: p = 0.007; α = 0.0077), and ADf vs. Cf (p < 0.0001; α = 0.0081). The estimated OR associated with ε4 was considerably higher in females ['ε4 dosage': 18.53 (6.18, 55.61); 'ε4 positive': 20.53 (6.80, 62.01)] than males ['ε4 dosage': 2.81 (1.36, 5.82); 'ε4 positive': 2.66 (1.14, 6.20).

### Analysis of gene-gene interactions

#### LD analysis

In the ADm group but not the Cm group, significant multi-locus LD at the modified FDR level was detected between *APOE *and lhcgr2 (p = 0.003; α = 0.0077; Table 2), while a number of pairs of loci exhibited marginally significant LD (p < 0.05; Table 2). Significant LD at the modified FDR level was also detected between *APOE *and lhcgr2 in Cf (p = 0.007; α = 0.0077; Table 2). A number of *LHCGR *loci in a number of different AD and control groups were found to be in significant LD with several *LHB *loci, especially lhb1 (Table 2).

**Table 2:** Loci exhibiting pairwise linkage disequilibrium at p <= 0.05. Bold-faced loci indicate a combination detected at the α = 0.05 level in an AD stratum but not in the corresponding control stratum. These multi-locus combinations were used as models in LR analyses.

	**Loci**	**D'**	**p-value**		**Loci**	**D'**	**p-value**
***AD males***	**lhcgr1/lhb1**	0.576	0.029	***Control males***	lhcgr1/lhb3	1.000	0.019
	lhcgr1/lhb5	0.388	0.020		*lhcgr1/lhb5*	0.494	0.033
	**lhcgr2/*APOE***	1.000	0.002^a^		*lhcgr4/APOE*	0.643	0.048
	**lhcgr5/lhb2**	0.305	0.023		*lhcgr5/APOE*	0.746	0.047
	**lhcgr5/lhb4**	0.293	0.033				
	**lhcgr5/lhb5**	0.335	0.028				
							
***AD females***	lhcgr2/*APOE*	0.520	0.032	***Control females***	lhcgr1/lhb1	0.415	0.004^b^
	**lhb3/*APOE***	0.718	<0.0001^b^		lhcgr1/*APOE*	0.281	0.031
					lhcgr2/*APOE*	0.315	0.007^b^
							
***All AD***	lhcgr1/lhb1	0.465	0.017	***All Control***	lhcgr1/lhb1	0.352	0.006^c^
	**lhb3/*APOE***	0.540	<0.0001^c^		lhcgr1/lhb2	0.419	0.021
					lhcgr2/*APOE*	0.265	0.044
							
***All males***	lhcgr1/lhb1	0.387	0.004^a^	***All females***	lhcgr1/lhb1	0.369	0.01^a^
	**lhcgr1/lhb2**	0.301	0.047		lhcgr2/*APOE*	0.294	0.019
	**lhcgr1/lhb3**	0.365	0.009^a^		lhb3/*APOE*	0.296	0.041
	**lhcgr1/lhb4**	0.312	0.038				
	**lhcgr1/lhb5**	0.458	0.001^a^				
	**lhcgr1/lhb6**	0.323	0.028				

^a ^significant at the corrected α = 0.0077 level (modified FDR) for the male dataset

^b ^significant at the corrected α = 0.0082 level (modified FDR) for the female dataset

^c ^significant at the corrected α = 0.0081 level (modified FDR) for the total dataset.

*APOE *and *LHB *are closely linked to one another (chromosomal region 19q13), separated by only 4.1 megabases. We took great care to ensure that any associations with AD observed in *LHB *were not the result of linkage with *APOE*. The only instance of significant LD between an *LHB *locus and *APOE *alone was found in the ADf and total AD groups (lhb3, p < 0.0001 for both groups; α = 0.0081 and α = 0.0082, respectively). It is difficult to interpret this result as an indication of LD that is simply due to physical proximity of the loci, since none of the other *LHB *loci exhibited even marginally significant LD with *APOE*. lhb3 was not identified as a main effect, nor as a component of any other significant interactions.

#### MDR analysis

MDR models were deemed significant when they met the *a priori *significance criteria described in Methods. For the AD vs. C comparison, one multi-locus combination was significantly associated with AD: lhcgr1/lhcgr2/*APOE *was selected as the best model in 6 of 10 cross-validation (CV) runs and produced a training accuracy of >0.5 in 9 of 10 CV runs. Two multi-locus models exhibited significant association with AD in the ADm vs. Cm comparison: lhcgr2/*APOE *(8 of 10 CVs, >0.5 training accuracy in 7 of 10 CVs) and lhcgr2/lhcgr5/*APOE *(7 of 10 CVs, >0.5 training accuracy in 9 of 10 CVs). One multi-locus model, lhb5/*APOE *(5 out of 10 CVs, >0.5 training accuracy in 10 of 10 CVs), was selected as significantly associated with AD upon comparison of the ADf and Cf datasets. No significant gene-gene interactions were detected using *APOE*-free datasets.

#### LR analysis

**Figure 2:**
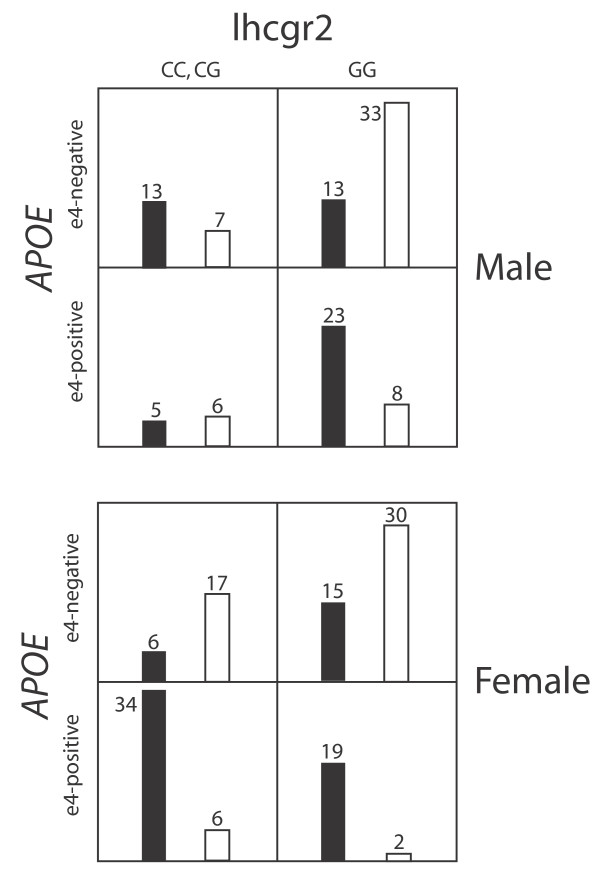
**Risk associated with ε4 allele is negated in male, lhcgr2 heterozygotes**. Contingency table illustrating the relative frequencies of lhcgr2/*APOE *genotypes in males and females (black = AD; white = Control).

LR analysis of the interactions suggested by LD and MDR analyses supported two interactions as marginally significant (lhcgr5/lhb2, both co-dominant, p = 0.05; lhcgr5/lhb4, both co-dominant, p = 0.041) and one interaction (lhcgr2/*APOE*) as significant at the modified FDR level. The most significant combination of lhcgr2 and *APOE *genotype models was the ε4-positive model of *APOE *and the C-dominant model of lhcgr2 (p = 0.003). As illustrated in Figure 2, the OR associated with this interaction [0.06 (0.01, 0.38)], represents a marked decrease in risk of AD for males who possess 1 or 2 'C-alleles' for lhcgr2 and are ε4-positive. Upon identification of this interaction we sequenced lhcgr2 and *APOE *in 10 additional male (5C and 5 AD) and 30 additional female (6C and 24 AD) samples. Use of the augmented datasets in LR analysis did not change the p-value or OR associated with the male-specific interaction and the lhcgr2/*APOE *interaction was still insignificant amongst females (p = 0.871).

### Identification of a novel, missense mutation in *LHCGR*

**Figure 3:**
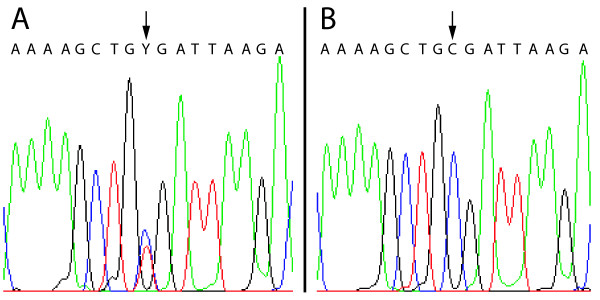
**Novel missense mutation in exon 11 of LHCGR**. (A) Chromatogram from one of two samples heterozygous for a novel C->T (Arg->Stop) missense mutation in codon 479 (exon 11) of *LHCGR*. (B) Chromatogram from a sample homozygous for 'C' at the same site.

Two males, one control and one AD, were heterozygous for a previously unreported C->T (Arg->Stop) missense mutation at the first position of codon 479 (exon 11) of *LHCGR *(Figure 3).

## Discussion

In general, our results suggest that putative associations should be treated with caution if they do not receive consistent support from biologically or statistically distinct analyses or are discovered using only one analytic method. Results of multiple analyses have the potential to strengthen support for disease association, point to alternate explanations of anomalous allele or genotype frequencies, or disabuse one of the notion that a particular polymorphism, unrelated to a disease of interest, plays a central role in its etiology.

### Multi-analytic approach to detection of gene-gene interaction

Studies that aim to identify genetic interactions are best served by the multi-analytic approach to data analysis practiced here and, for family-based data, by [30]. In the absence of biochemical evidence, putative genetic interactions are often difficult to accept intellectually. Multiple lines of statistical support, which represent the identification of a significant genetic interaction using different statistical and biological approaches, increase statistical and intellectual confidence in the biological reality of an interaction. In this study, LR and MDR provided distinct statistical/computational methods for the detection of significant genetic interactions, while LD analysis provided a distinct biological approach to the problem of interaction detection. A majority of AD-associated interactions reported here lack intra-study corroboration, as they were only identified by one analytical method (Table 3). As such, these interactions are not well supported. On the other hand, lhcgr2/*APOE *(the only interaction supported by all 3 analytic methods at the modified FDR level) is a better candidate for biochemical study. In addition, more support for lhcgr2/*APOE *is derived from the association of lhcgr2 with differences in AoO. The different analyses are not, of course, statistically independent of one another, as the same dataset is being analyzed in each case. Nevertheless, *because *they are not independent, failure to identify the same interaction using distinct, robust analytical methods seriously impugns the biological reality of a putative interaction.

**Table 3:** Distribution of significant associations between non-*APOE *polymorphisms and AD. LD = linkage disequilibrium, MDR = multi-dimensionality reduction, LR = logistic regression, AoO = age of onset, ●● = significant at the modified FDR α level, or, in the case of MDR, according to *a priori *significance criteria: for significant MDR results, the proportion of 10 CVs that identified this model as best and proportion of 10 CVs in which this model produced a training accuracy >0.5 are listed; ● = approaching significance (p <= 0.05, the experimentwise α). Note the consistent identification of lhcgr2/*APOE *as a significant interaction in males. CV = cross-validation.

**Loci**	**Dataset**	**χ^2^**	**AoO**	**LD**	**MDR**	**LR**
lhb2	♂	●				
lhcgr2	♂		●● (p = 0.001; α = 0.0077)			
lhcgr1/lhb1	♂			●		
lhcgr1/lhb5	♂			●		
lhcgr2/*APOE*	♂			●● (p = 0.002; α = 0.0077)	●● (0.8 CVs; 0.7 CVs)	●● (p = 0.003; α = 0.0077)
lhcgr5/lhb2	♂			●		●
lhcgr5/lhb4	♂			●		●
lhcgr5/lhb5	♂			●		
lhcgr2/lhcgr5/*APOE*	♂				●● (0.7 CVs; 0.9 CVs)	
lhcgr2/*APOE*	♀			●		
lhb3/*APOE*	♀			●● (p < 0.0001; α = 0.0081)		
lhb5/*APOE*	♀				●● (0.5 CVs; 1.0 CVs)	
lhcgr1/lhb1	Total			●		
lhb3/*APOE*	Total			●● (p < 0.0001; α = 0.0082)		
lhcgr1/lhcgr2/*APOE*	Total				●● (0.6 CVs; 0.9 CVs)	

### APOE, LH signaling, gender-specific effects, and AD

Polymorphisms of other HPG-axis proteins (estrogen receptors α and β) are associated with increased susceptibility to AD in women [10,11]. In this respect, prophylactic and therapeutic use of natural estrogen (17β-estradiol) has been consistently demonstrated to delay disease progression in women [11]. As LH signaling is directly involved with reproduction, produces gender-specific physiological and anatomical endpoints, and has been associated with AD, LH and its receptor also present good candidates for gender-specific associations with disease. The male-specific nature of the significant lhcgr2/*APOE *interaction identified in our analyses (Table 3), and its relation to *APOE *genotype, is important. Gender is thought to interact with *APOE *genotype [31,32], and our data support the hypothesis that the ε4 allele is more strongly associated with female than male AD: 49% of ADm and 70% of ADf were ε4 positive. If ε4 does provide less explanatory power in males, it is logical to suggest that male-specific risk factors for AD do exist. Indeed, one recent study identified an association between number of CAG repeats in the androgen receptor and AD [33]. Should further sampling corroborate the male-specific association with AD of lhcgr2/*APOE *(Table 3), it will become imperative to elucidate the biochemical basis of this gender bias (see discussion of the lhcgr2 site below). While gender-specific hormonal fluctuations, namely the rise in LH serum levels following menopause, have been suggested to account for the disproportionately greater number of females who acquire AD [34,35], the idea that common differences in the actual sequence and structure of LHβ and its receptor might only affect males is intriguing. We can exclude issues related to genetic or trait heterogeneity as explaining our results since all scored loci exhibit HWE in males and the dataset is consistent with past sampling of *APOE *genotype in males. ε2, ε3, and ε4 allele frequencies among affected males in our study were 0.03, 0.66, and 0.32, respectively, which are not obviously different from those reported in a meta-analysis of 5107 case-control Caucasian AD subjects (4): 0.039 (ε2), 0.594 (ε3), and 0.367 (ε4). 49% of ADm and 70% of ADf were ε4-positive in our study, which is strikingly similar to the 46.6% ε4-postive males and 72% ε4-positive females reported in a previous paper suggesting interaction between gender and *APOE *genotype (36). Additionally, neither mean age (83.34 +/- 5.14 yrs in males, 83.34 +/- 5.58 yrs in females; p = 1.00) nor mean AoO (79.18 +/- 3.47 yrs in ADm and 80.26 +/- 5.07 yrs in ADf; p = 0.22) were significantly different in males and females. Documented instances of alcohol and drug use, cardiovascular disease, and stroke were equally rare in males and females of our cohort.

Despite strong support for the association between lhcgr2/*APOE *and AD, the details of the interaction are paradoxical. While the ε4 allele carried significant risk of AD in males of our dataset (p = 0.007), males who carried 1 or 2 C-alleles at the lhcgr2 locus and were ε4 positive had a significantly reduced risk of AD (odds ratio: 0.04; 95% confidence level: 0.01, 0.32; p = 0.002). As both increased LH levels [17] and the *APOE *ε4 allele [36,37] are associated with increased Aβ deposition, and neurosteroid production, it is reasonable to suggest that LH signaling and *APOE *genotype interact to modify an individual's susceptibility to AD. The significant decrease in risk of AD observed in ε4-positive males with 1 or 2 lhcgr2 C-alleles lends support to the possibility that lhcgr2-dependent alternative splicing of *LHCGR *pre-mRNA leads to isoforms of LHCGR that are functionally distinct (see below), or that lhcgr2 is part of an intron-derived microRNA (miRNA) capable of regulating *APOE *mRNA translation (see below). Despite the absence of empirical evidence to support the existence of unreported LHCGR isoforms or miRNAs derived from *LHCGR *introns, our data do support a complex, gender-specific interaction between *LHCGR *and *APOE*. Of note, LH elevates APOE secretion from cultured interstitial cells, thereby increasing the availability of cholesterol for sex hormone production [38]. LH also increases low-density-lipoprotein receptor-related protein expression in granulose cells [39]. If such processes occur in the brain, then the protective effects of an *LHCGR*-*APOE *interaction in males may be mediated via increased neurosteroid production and the male-specific nature might be explained by differential protective effects of androgens and estrogens.

### Intronic polymorphisms and lhcgr2 as a cryptic splice site or intron-derived miRNA

**Figure 4:**
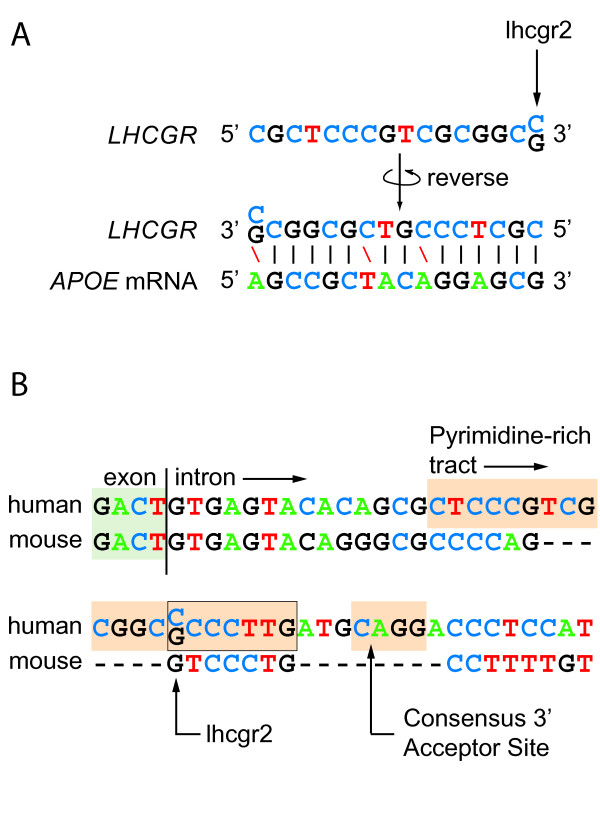
**lhcgr2 shares similarity with consensus 3' acceptor splice sites and ApoE mRNA**. (A) lhcgr2 as a potential miRNA that regulates the expression of ApoE. The reversed *LHCGR *sequence is complimentary to a fragment of *APOE *mRNA at 13 out of 16 sites. (B) Pairwise sequence alignment of a portion of LHR intron 1 in human and mouse (*Mus musculus*). The human sequence includes lhr2 (rs4073366) and bears sequence features characteristic of 3' acceptor splice sites.

The most significant interaction associated with AD in this study includes lhcgr2, a polymorphism located in intron 1 of *LHCGR*. Intronic polymorphisms are frequently implicated in increased disease susceptibility [11,40,41] and intronic mutations have the potential to alter protein sequence dramatically. An intronic mutation can cause the disarrangement of an existing splice site or introduce a cryptic splice site, resulting in the addition/removal of hundreds of amino acids or premature termination of translation. A second mechanism by which intronic mutations might affect disease etiology is through their effect on the miRNAs that may be derived from the mutated intron. ~10–30% of spliced intronic material is exported to the cytoplasm, where it has the potential to act as an miRNA and alter protein expression [42]. It follows that mutation of intronic sequence could decrease or increase the complementarity of an intron-derived miRNA to its target mRNA, thereby causing a relative decrease or increase in expression of the encoded protein. Indeed the 15 bases immediately upstream of lhcgr2 are complementary to *APOE *mRNA (Figure 4A), indicating this polymorphism may cap an intron-derived miRNA.

Examination of the sequence surrounding lhcgr2 and alignment of human and mouse (*Mus musculus*) *LHCGR *intron 1 indicate lhcgr2 may be located within a cryptic 3' acceptor splice site (Figure 4B). Acceptor splice sites are characterized by two conserved sequence patterns: a pyrimidine-rich sequence, known as the polypyrimidine tract, and the proximate terminal 'AG' of the intron [43]. Although the length of the polypyrimidine tract and its distance from the end of the intron are variable, the terminal 'AG' is invariant [43,44]. Polymorphism lhcgr2 is located within a pyrimidine-rich region of intron 1 (Figure 4B). The lhcgr2 'C-allele' increases the CT-content of the surrounding 19 nucleotides to 79%, and, more locally, a 'C' at lhcgr2 forms a contiguous sequence of 7 Cs and Ts. Four bases downstream of this pyrimidine-rich tract is a 3' acceptor site consensus sequence, CAGG. Absence of a homologous sequence in mouse *LHCGR *intron 1 may indicate that retention of this sequence in *Homo sapiens *is due to its potential use in alternative splicing. To investigate the degree to which a 'C' at lhcgr2 increases the similarity of the local sequence to human acceptor sites in general, lhcgr2 'C' and 'G' alleles were entered in the online splice site prediction programs GENIO/splice and SpliceScan [45]. Both programs identified lhcgr2 as a potential acceptor splice site and, based on the programs' output scores, indicated that a 'C' at the site does increase its similarity to stereotyped 3' acceptor splice sites.

### Linkage disequilibrium between lhcgr1 and multiple LHB loci

**Figure 5:**
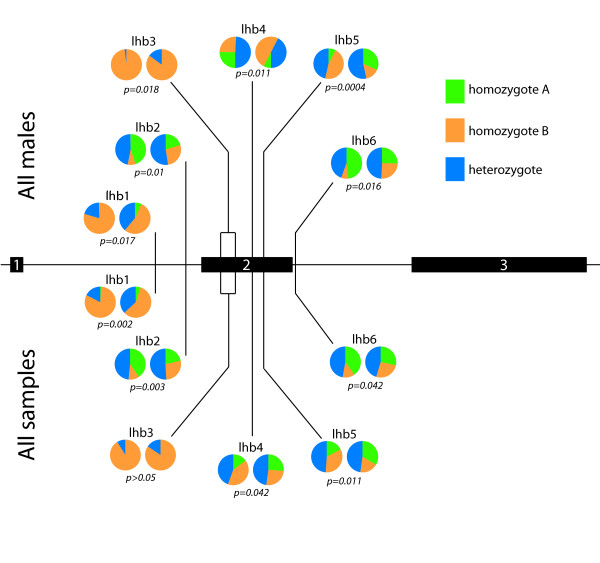
**Significant linkage disequilibrium between lhcgr1 and multiple *LHB *loci**. In the schematic of *LHB*, exons are represented as black boxes and the positions of the 7 *LHB *polymorphisms scored are indicated by vertical lines. lhb3 is composed of two SNPs, which are in complete LD with each other. For each polymorphism, genotype frequencies are represented in the form of two pie charts, where the left chart represents genotype frequencies among individuals lacking an LQ-insert allele in exon 1 of *LHCGR *(lhcgr1) and the right chart represents genotype frequencies among individuals possessing 1 or 2 LQ-insert alleles. Frequencies above the graphs correspond to AD males, while frequencies below the graph correspond to *all *individuals sampled in this study. * = significant difference (p < 0.05), ** = highly significant difference (p < 0.01).

LD between lhcgr1 and LHB loci was commonly detected in several AD and C datasets (Table 2), including 6 instances in the 'All male' dataset. The frequent identification of LD between lhcgr1 and *LHB *loci indicates that specific *LHB *haplotypes are found most frequently in individuals that are hetero- or homozygous for the LQ-insertion at lhcgr1. We questioned whether our data supported the idea that the LQ-insertion at lhcgr1 is, in general, in LD with *LHB *loci. To examine this possibility, we compared the frequency of genotypes at all 6 *LHB *loci between individuals with no LQ-inserts and those with 1 or 2 LQ-inserts (Figure 5). In both the group of all males *and *the group of all samples, χ^2^-tests revealed that the genotype counts of a majority of *LHB *loci were markedly different from one another in the LQ-insert and no-LQ-insert strata (Figure 5). This suggests: (1) a non-random force, such as selection, is driving the non-random association of LQ-insert alleles with specific *LHB *haplotypes in the North American Caucasian population – i.e., certain variants of *LHB *may be better suited to the LQ-insert variant of *LHCGR*; (2) given the extent of LD between lhcgr1 and *LHB *loci in C and AD samples, instances of significant LD between lhcgr1 and *LHB *loci in AD samples are not indicative of AD-associated interactions.

## Methods

### Case-control setup

The National Cell Repository for Alzheimer's Disease (NCRAD; University of Indiana, Bloomington, IN) provided total DNA samples from 200 control (negative for AD and other neurodegenerative diseases, 50 male and 50 female) and late-onset AD patients (negative for other neurodegenerative diseases, 50 male and 50 female). All samples were obtained from North American Caucasian subjects. All samples were derived from individuals >= 75 years of age, and all AD samples were acquired from individuals whose AoO was = 75. Mean age of the control group was 84.73 w 4.61 years, while mean age of the AD group was 81.95 ± 5.69 years. Among the AD samples, AoO was 79.18 ± 3.47 years for males and 80.26 ± 5.07 years for females. DNA was obtained from 40 additional samples (10 male samples – 5 control, 5 AD and 30 female samples – 6 control, 24 AD) to test for significant interaction between *APOE *and an *LHCGR *locus (lhcgr2, see below). Direct sequencing of *APOE*, *LHB *(promoter, signal, and coding regions), and *LHCGR *(exons 1, 10, and 11) was performed using the primer pairs listed in Table 4. Cycle sequencing products were run on an ABI 3730 XL DNA analyzer at the University of Wisconsin Biotechnology Center (Madison, WI) and the resultant chromatograms were analyzed with FinchTV v1.4 (Geospiza, Seattle, WA). This study was carried out with IRB approval from the Health Sciences Institutional Review Board of the University of Wisconsin.

**Table 4:** Primer pairs used to amplify portions of *APOE*, *LHB*, and *LHCGR*.

**Fragment**	**Sequence**
*LHB *5' [50]	F: 5'-GTTACCCCAGGCATCCTATC-3' R: 5'-CCATTCCCCAACCGCAGG-3'
*LHB *3' [50]	F: 5'-GGTCCTGAATAGGAGATGCCA-3' R: 5'-CGGGGTGTCAGGGCTCCA-3'
*LHCGR *exon 1 [51]	F: 5'-CACTCAGAGGCCGTCCAAG-3' R: 5'-GGAGGGAAGGTGGCATAGAG-3'
*LHCGR *exon 10 [51]	F: 5'-ACAGTCAGGTTTAGCCTGAA-3' R: 5'-CTTCTGAGTTTCCTTGCATG-3'
*LHCGR *exon 11 (5') [52]	F: 5'-CAGAAAATCCCTTACCTCAAGC-3' R: 5'-GGTTTAAGAACAATTCAATAATGCAG-3'

### Data analysis

Similar to the analytic paradigm suggested by [30] for *family-based *data, we chose to analyze our case-control dataset using an array of analytic methods, testing for interactive as well as main effects, and treating the convergence of results from distinct analyses as the best evidence of association. Allele and genotype counts were used in the following analyses: (1) χ^2 ^tests of allele and genotype counts to test for main effects of individual polymorphisms; (2) tests of each locus for Hardy-Weinberg Equilibrium (HWE); (3) tests of combinations of two or three loci for linkage disequilibrium (LD); (4) tests for gene-gene interactions using multifactor dimensionality reduction (MDR); (5) tests for interactions using logistic regression (LR), and; (6) tests for association of polymorphisms with age of onset using one-way ANOVA. Additionally, to control for heterogeneity we stratified the dataset according to gender and applied the same 6 analyses. Finally, for each bi-allele locus, four genotype models were analyzed in tests for interactive effects: co-dominant, allele 1 dominant, allele 2 dominant, and over-dominant. This schema enabled us to: (1) address the possibility of heterozygote advantage, and; (2) test both alleles for dominance, as we had no *a priori *knowledge of which allele might carry risk.

For each sample, genotype and demographic data were entered into a MySQL relational database, enabling the quick identification of samples meeting an array of criteria. *APOE *genotype and 14 previously reported polymorphisms of *LHB *and *LHCGR *were scored. For each polymorphism, allele and genotype frequencies of the AD and control groups were calculated. Additionally, both groups were stratified by gender and gender-specific allele and genotype frequencies were calculated. Four separate genotype models were used in tests for main and interactive effects of bi-allele loci. For example, the following models would be used for a locus that varied between alleles B and b: (1) co-dominant (BB vs. Bb vs. bb); (2) B dominant [(BB + Bb) vs. bb]; (3) b dominant [BB vs. (Bb + bb)], and; (4) over-dominant [(BB + bb) vs. Bb]. For the tri-allele *APOE*, an 'ε4 dosage' model (genotypes grouped by the number of ε4 alleles) and an 'ε4 positive' model (ε4 allele present or not) were used in analyses.

The program Genetic Data Analysis (GDA) [46] was used to test each polymorphic locus for HWE. Minitab [47] was used to test for the association of individual polymorphisms with AD (χ^2 ^tests of allele and genotype counts) and AoO in the AD groups (ANOVA). In all tests for LD, genotypes were preserved in order to prevent significant deviations from HWE at a single locus from contributing to the measure of LD. We considered a number of theoretical issues when designing our analytical approach to detect gene-gene interactions (see Supplementary Information) and ultimately chose the combination of LD, MDR and LR analyses. Pairwise tests for LD were performed using the program PyPop, where the p-value reported here is derived from the difference between the likelihood of the inferred haplotype frequencies and the likelihood of the data if the two loci are assumed to be in linkage equilibrium [48]. We reported the D' measure of LD, as this is an intuitive metric that represents the estimated proportion of maximum possible LD exhibited by the sample data. MDR was performed using MDR Software [49], which output the best 1-, 2-, 3-, and 4-factor models for a given dataset. 10-fold cross-validation was used. Given the weight *APOE *carries as a single factor, MDR was also run using *APOE*-free datasets in order to detect any interactions that did not include *APOE*. An interaction model was considered significant if it was selected as the best model in 5 or more of the CV runs and exhibited a testing accuracy of >0.5 in 7 or more CV runs. Pairs of loci exhibiting significant LD (p <= 0.02) and significant multi-locus models discovered using MDR were input as disease models in LR analyses performed in [47]. This form of LR model selection was necessary, as a lack of several multi-locus combinations made backward model selection impossible and the lack of significant main effects in most loci studied made forward model selection impractical.

To account for multiple tests, testwise α levels were corrected using modified FDR (see Supplementary Information). Because a multi-locus combination was only tested with LR if LD and/or MDR analyses were suggestive of its association with AD, only a subset of the total array of possible LR tests were actually performed and the total, male, and female datasets were subjected to a different total number of tests: 182, 252, and 190 tests, resulting in modified FDR α levels of 0.0082, 0.0077, and 0.0081, respectively.

## Conclusion

We report the discovery of a genetic interaction between APOE and LHCGR alleles that is associated with a significantly decreased risk for AD in males. The biochemical basis for this interaction is uncertain, although alternative mRNA splicing and intron-derived miRNA regulation are hypothesized as distinct possibilities. Our results emphasize the importance of testing for gene-gene interactions in studies of complex disease. We suggest that the best evidence for epistasis is obtained when multiple analyses, distinct in their biological or statistical basis, converge on a positive result.

## Appendix

### Methodological strategies for the detection of gene-gene interactions

We searched for genetic association of single loci with AD using standard χ^2 ^and HWE tests and considered that subsequent tests for gene-gene interactions may identify interactions whose loci may or may not produce significant main effects on their own. There are a number of analytical issues to consider when searching for multiple interacting genetic (or environmental) factors associated with a disease of complex etiology. For one, genetic or trait heterogeneity among the samples (e.g., AD samples from males or females, or, with or without hypertension) has the potential to confound data analysis. Stratification of the dataset is the most straightforward method used to control for heterogeneity. For example, one might examine the effects of *APOE *heterogeneity by splitting control and AD groups into ε4 positive and negative strata and asking: Is the frequency of an SNP of interest significantly higher in ε4-positive AD samples than in ε4-positive controls, but *not *in the comparable ε4-negative comparison? If so, the data suggest an interactive effect between the SNP and ε4. Significantly, a non-stratified comparison of AD and control groups in such a case might lead a researcher to conclude the SNP has no association with AD, or, conversely, that the SNP is associated with AD in ε4 positive *and *negative individuals. Though more mathematical methods to control for heterogeneity exist, the majority of them are not applicable to case-control data.

In studying the genetics of a complex disease, it is important to consider the possibility that gene-gene or gene-environment interactions produce interactive effects that provide significant explanatory power, even in the absence of single factor, main effects [53]. A number of methods allow researchers to test for gene-gene interactions using case-control data. A traditional method is logistic regression (LR), which, given a dataset, models the probability of a discrete outcome (in our case, AD or not) on *n *factors and their interactions, each qualified by a coefficient estimated using Maximum Likelihood Estimation. Attractively, LR produces an Odds Ratio (OR), which provides the researcher with an intuitive measure of how a particular array of genetic and/or environmental factors affects the likelihood of developing the disease. The major shortcoming of LR is the so-called 'curse of dimensionality', which refers to poor coefficient estimation resulting from too few or no examples of various multi-factor combinations in the dataset if sample size is too small or number of factors too large [54].

An alternative to LR analysis for the detection of interactions is multifactor dimensionality reduction (MDR) [55], which is advantageous for several reasons. A chief, practical advantage is that the researcher can easily test disease models that include interaction terms whose components lack significant main effects. This is critical, since it is increasingly apparent that genetic interactions, in the absence of main effects, frequently contribute to the susceptibility of an individual to complex diseases like AD [56]. Also important, MDR is not "cursed" by dimensionality: the method is robust even when the input dataset lacks examples of various, multi-factor combinations. Finally, unlike LR, MDR analysis automatically measures the predictive accuracy and validity of a selected model through partitioning of the original dataset into training and testing subsets [57,58].

Another approach to gene-gene interaction detection is to calculate linkage disequilibrium (LD) amongst combinations of the loci under investigation. Significant LD among 2 or more loci indicates non-random segregation of the loci in question, which implies that at least one multi-allele combination at these loci is overrepresented. Logically, any multi-allele combination enriched in the case but not the control group is considered to contribute increased susceptibility to the disease, and is reflected by a measurement of significant LD in the case group only [59]. Williams et al. [60] used this approach in a study of polymorphisms associated with hypertension, discovering 16 combinations of 7 loci in 5 genes that exhibited significant LD in the case group only. Significantly, none of these loci were associated with a main effect on hypertension, indicating LD analysis has the ability to detect potentially significant gene-gene interactions in the absence of main effects.

## Authors' contributions

RJH designed the study, collected the samples, performed the genotyping, analyzed the data, performed the statistical analyses and drafted the manuscript. MRA performed genotyping and data analysis. SVM and RLB helped to draft the manuscript. CEG, SCJ and SA provided DNA samples from confirmed AD and control individuals that they characterized. CSA conceived the study, participated in its design and coordination and helped to draft the manuscript.

## Pre-publication history

The pre-publication history for this paper can be accessed here:


